# Early Post-operative ECG Changes as a Predictor of Post-pericardiotomy Syndrome Following Atrial Septal Defect Repair

**DOI:** 10.1007/s00246-024-03464-2

**Published:** 2024-04-06

**Authors:** Kristen Hyberg, Iqbal El-Assaad, Wei Liu, Iqbal El-Assaad, Orkun Baloglu, Howard Heching, William Hanna

**Affiliations:** 1 Department of Pediatric Critical Care Medicine, Cleveland Clinic Children’s, 9500 Euclid Avenue, Cleveland, OH 44195 USA; 2 Department of Quantitative Health Sciences, Cleveland Clinic Children’s, 9500 Euclid Avenue, Cleveland, OH 44195 USA; 3 Department of Pediatric Cardiology, Cleveland Clinic Children’s, 9500 Euclid Avenue, Cleveland, OH 44195 USA

**Keywords:** Atrial septal defects, Post-pericardiotomy syndrome, ECG, Post-operative

## Abstract

To identify risk factors associated with post-pericardiotomy syndrome (PPS) in patients undergoing surgical repair of atrial septal defects (ASD). A single-center retrospective study. Tertiary academic hospital. Included were patients of all ages who underwent surgical ASD repair, while exclusion criteria included the absence of post-operative electrocardiogram (ECG), lack of follow-up post-discharge and factors hindering ECG interpretation. Demographic and clinical data, including ECG changes indicative of pericardial inflammation, were collected. The primary outcome measure was the development of PPS, determined based on the standardized European Society of Cardiology (ESC) criteria. Among 190 patients who underwent surgical ASD repair, 154 (81%) met the inclusion criteria. Of these, 25 (16%)in total developed PPS, of which 60% were ≥ 18 years of age and 56% female. Significant associations relating both early ECG changes and pre-discharge pericardial effusion with subsequent occurrence of PPS were found in both univariate and multivariate analyses. The study establishes correlations of both early post-operative ECG changes indicative of inflammation and pre-discharge pericardial effusion with subsequent occurrence of PPS in patients undergoing surgical ASD repair. Both utilizing the standardized ESC definition of PPS and incorporating a physician-validated ECG evaluation strengthened the methodologic approach in establishing these relationships. The results also highlight the importance of considering age as a potential risk factor for PPS. Further research is needed to validate these findings and explore additional risk factors predicting early identification and management of patients at high risk for PPS following surgical ASD repairs.

## Introduction

Post-pericardiotomy syndrome (PPS) is a common complication following cardiac surgeries, affecting 10–40% of patients. PPS can result in increased hospitalization with the most serious complications including cardiac tamponade and constrictive pericarditis [1–6]. Although first described in the 1950’s, there was not a unified definition of PPS prior to 2015, when the European Society of Cardiology (ESC) proposed a standardized diagnostic criterion, allowing for a more uniform approach to its study [1, 3, 7]. This definition established PPS when patients qualified for 2 of 5 criteria: fever, pericarditic or pleuritic chest pain, pericardial or pleural friction rub, pericardial effusion, and pleural effusion with elevated C-reactive protein (CRP). Patients can develop these findings from a few days up to 3 months post-surgery.

While the exact pathogenesis of PPS is not clear, the current belief is that PPS is an immune mediated inflammatory condition triggered by an insult to the pericardium. This is supported by a rise in inflammatory markers with PPS, such as ESR and CRP, and associations found with underlying autoimmune disorders. Cardiac surgery may induce this process in several different ways, including ischemia from cardiopulmonary bypass, direct trauma from surgery, and irritating blood remaining in the pericardium [6–8].

Past studies have attempted to identify risk factors for PPS despite these methodologic difficulties. While there are conflicting data, the risk factors that have been associated with PPS include type of cardiac surgery (atrial septal defect closure in particular), younger age, need for blood transfusion, and elevation in inflammatory biomarkers [3, 5, 6, 8–15].Utilizing the standardized ESC definition of PPS, we sought to further characterize early risk factors associated with PPS development in perioperative patients with surgically repaired ASDs. Among other predictor variables, we hypothesized that signs of pericardial inflammation on Electrocardiogram (ECG) when studied serially over the first 72 h of post-operative admission will be associated with the development of PPS, a finding with a plausible physiologic basis that has been inconsistently studied in available literature. Unique to this study as well is the epidemiologic focus including both adult and pediatric patients within a single center and the methodologic focus on ensuring ECG changes be validated via blinded cardiologist interpretation.

## Methods

### Study Design and Setting

This was a single-center retrospective study that was approved by our institutional review board. We performed an observational chart review of all patients who underwent surgical repair of ASDs between January 2012 and July 2021. Exclusion criteria included patients without ECG data postoperatively and those without at least one follow-up visit one month or later after their surgical repair. Patients were also excluded if they had single ventricle physiology, required cardiac pacing, or had a bundle branch block making ECG interpretation for ST segment changes potentially unreliable.

### Data Collection and Definitions

The study participants were identified from the electronic medical record system based on primary diagnosis of surgical ASD repair. Charts that met inclusion criteria and did not meet exclusion criteria were then reviewed. Study data were entered into Research Electronic Data Capture (REDCap®).

Demographic data collected included patient’s age, sex, blood type, and allergies. Other data collected included type of repair, blood product received, cross clamp time, bypass time, amount of chest tube drainage, use of steroids perioperative, NSAID use, creatine, highest white blood cell count, platelet count, the presence of pericardial effusion prior to discharge, and length of stay.

Our primary predictor variable was post-operative ECG changes indicative of pericardial inflammation during the post-operative period. A pediatric electrophysiologist blinded to post-operative PPS status reviewed the patient's ECGs for signs of inflammation based on the pattern of ST elevation as well as presence or absence of PR segment depression and in comparison to the preoperative ECG. This assessment was coded dichotomously based upon a pattern consistent or not with pericarditis, including a diffuse elevation pattern or ST segment elevations in multiple leads not present on preoperative ECGs. ST elevation was defined as at least > 1 mm in limb leads and > 2 mm in precordial leads. If ST elevation existed preoperatively and the pattern was similar post operatively, the ECGs were considered to represent early repolarization and coded as negative. Patients with complete right bundle branch block were excluded due to the inability to accurately evaluate repolarization changes in the setting of depolarization abnormality.

### Outcome Measures

Our primary outcome measure was the development of PPS. An independent reviewer reviewed patient charts and made this determination based on European Society of Cardiology criteria, which includes fever without alternate cause, pleuritic chest pain, evidence of pericardial effusion, pleural effusion with elevated CRP, and/or presence of pleural or pericardial rub. The onset of PPS was defined as occurring between 72 h and 3 months following the cardiac operation.

### Statistical Analysis

Descriptive statistics were used to summarize the data, presenting medians, quartiles, means, and standard deviations for continuous variables, and counts and percentages for categorical variables. Wilcoxon rank-sum or two-sample t-tests were used for comparisons of demographic and clinical characteristics between study groups for continuous and ordinal variables, while Chi-square or Fisher's exact tests were used for categorical variables.

Both primary predictor (ECG changes) and outcome (PPS development) variables were treated as dichotomous variables, and significance was assessed using Chi-square testing or Fisher's exact test, with a p-value < 0.05 considered significant. For secondary outcomes, Chi-square or Fisher's exact tests were used for unadjusted analyses, and logistic regression was employed for adjusted analyses. All tests were two-tailed, and a significance level of 0.05 was used. Statistical analyses were performed using SAS 9.4 software (SAS Institute, Cary, NC).

## Results

**Fig. 1:**
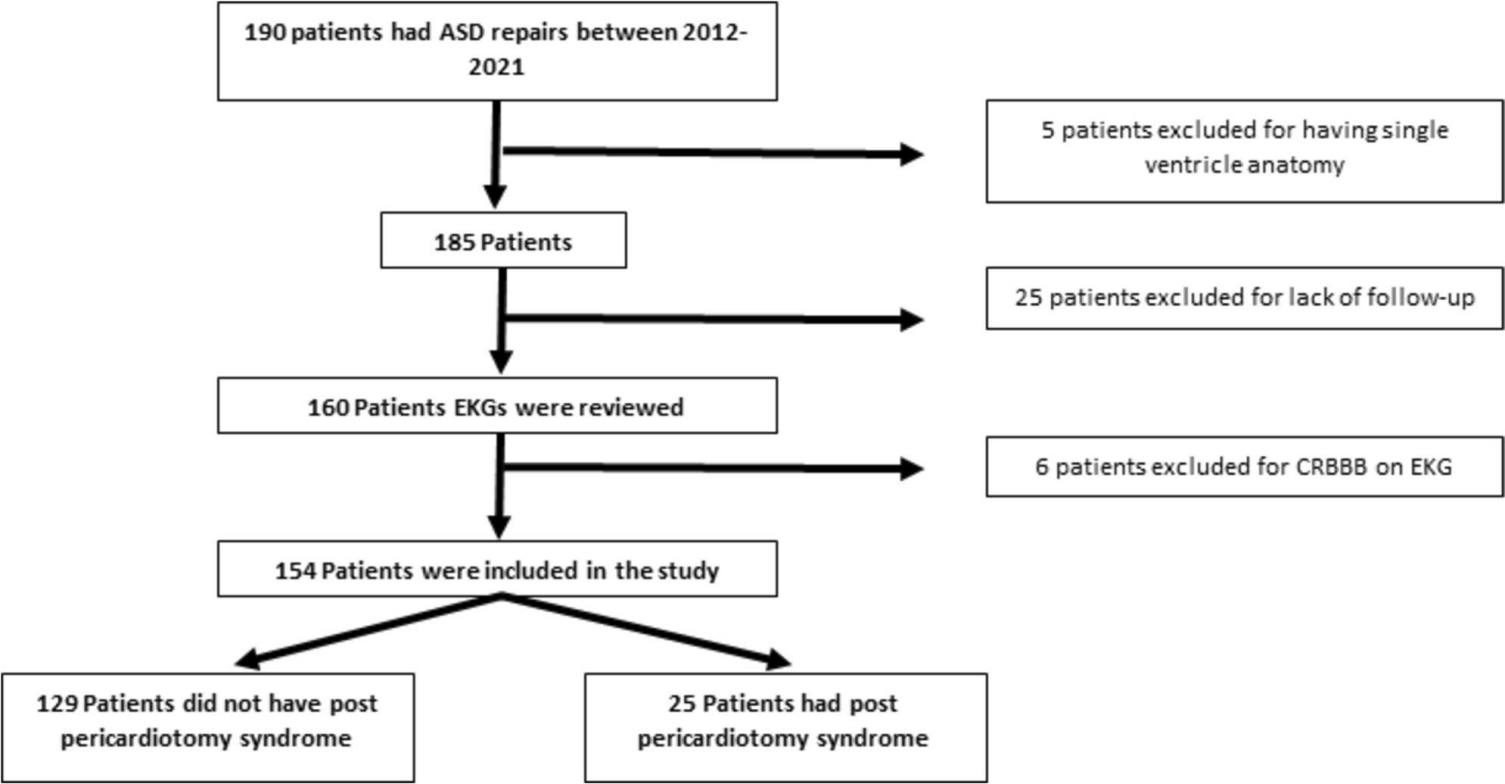
Inclusion and Exclusion criteria

A total of 190 patients underwent surgical ASD repair between January 2012 and July 2021. Among them, 5 (2.6%) patients were excluded due to single ventricle anatomy, 25 (13.2%) patients were excluded due to a lack of follow-up, and 6(3.2%) patients were excluded due to the presence of complete right bundle branch block on ECG, Fig. 1.

The final analysis included 154 patients, out of which 25 (16.2%) were positive for PPS (Fig. 1). In the non-PPS group, 38.8% of patients were adults comparing with 60.0% of adult patients in the PPS group (p = 0.049), with median ages in non-PPS group of 10 years [4] comparing with 22 years [13] in the PPS group (*p* = 0.03). No significant differences existed between gender in the two groups (61.2% vs 56.0% female between non-PPS and PPS groups, respectively (*p* = 0.62)).

Compared to patients who developed PPS, non-PPS patients had similar post-operative lengths of stay (4 days vs. 4 days, p = 0.47), chest tube drainage (8.9 mL/kg vs 8.8 mL/kg, *p* = 0.65), WBC (11.3 vs 11.9, *p* = 0.73), steroid administration (13 (52%) vs 54 (34.9%), *p* = 0.11) or blood product administration (3 (12%) vs 27 (20.9%), *p* = 0.30) (Table 1).

**Table 1:** Post Op characteristics by PPS

	Overall (N = 154)		No PPS (N = 129)		PPS (N = 25)		
Factor	N	Statistics	N	Statistics	N	Statistics	p-value
LOS (days)	154	4.0 [3.0, 6.0]	129	4.0 [3.0, 6.0]	25	4.0 [3.0, 6.0]	0.47^b^
ASD Closure	154		129		25		0.13^d^
Primary		62 (40.3)		53 (41.1)		9 (36.0)	
GorTex Patch		4 (2.6)		2 (1.6)		2 (8.0)	
CorMatrix Patch		2 (1.3)		1 (0.78)		1 (4.0)	
Pericardial Patch		86 (55.8)		73 (56.6)		13 (52.0)	
Bypass Time (min)	154	43.0 [31.0, 61.0]	129	44.0 [28.0, 65.0]	25	39.0 [34.0, 55.0]	0.57^b^
Cross-clamp Time (min)	153	30.0 [18.0, 43.0]	128	32.0 [17.5, 45.0]	25	27.0 [19.0, 34.0]	0.40^b^
Steroids in OR	154	58 (37.7)	129	45 (34.9)	25	13 (52.0)	0.11^c^
Amount of Chest tube drainage (mL/kg) total	154	8.9 [5.1, 16.3]	129	8.8 [5.2, 17.9]	25	8.9 [4.5, 12.7]	0.57^b^
Received blood products	154	30 (19.5)	129	27 (20.9)	25	3 (12.0)	0.41^d^
Initial WBC (k/ul)	123	11.4 [9.1, 14.2]	106	11.5 [9.1, 14.1]	17	11.1 [9.2, 14.7]	0.83^b^
Initial hemoglobin (g/dl)	123	10.9 [9.9, 12.2]	106	10.9 [9.8, 12.3]	17	11.0 [10.1, 12.1]	0.77^b^
Platelet count (k/ul)	123	165.0 [134.0, 204.0]	106	165.5 [134.0, 209.0]	17	162.0 [135.0, 193.0]	0.85^b^
Highest WBC (k/ul)	122	11.8 [9.6, 14.7]	105	11.9 [9.5, 14.7]	17	11.3 [11.0, 14.7]	0.56^b^
Pericardial effusion present prior to discharge	128	57 (44.5)	106	41 (38.7)	22	16 (72.7)	0.003^c^
Size of Effusion	57		41		16		0.16^b^
Trivial		36 (63.2)		28 (68.3)		8 (50.0)	
Mild		18 (31.6)		12 (29.3)		6 (37.5)	
Moderate		3 (5.3)		1 (2.4)		2 (12.5)	

Statistics presented as Median [P25, P75], N (column %). p-values: b = Wilcoxon Rank Sum test, c = Pearson's chi-square test, d = Fisher's Exact test

**Fig. 2:**
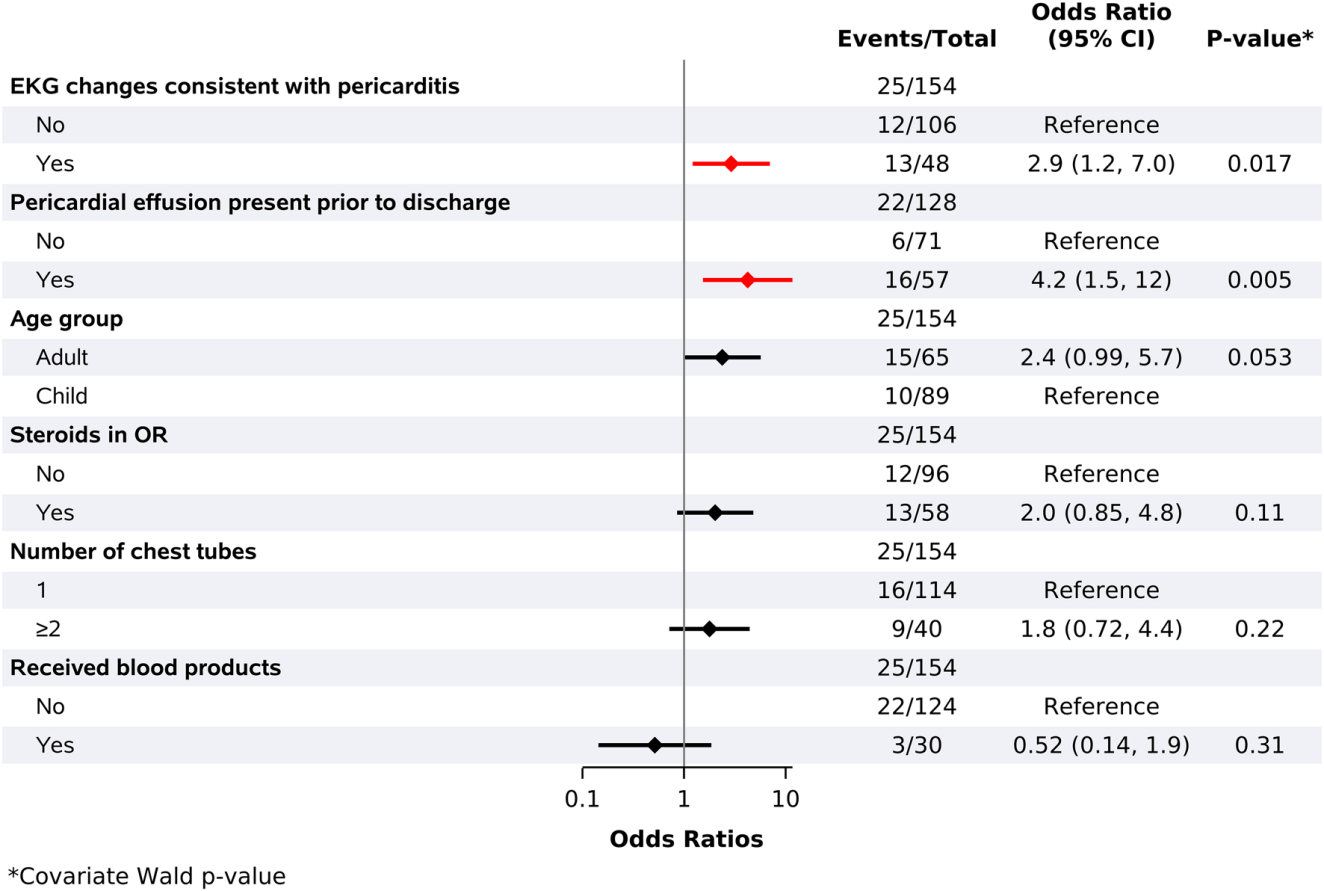
Univariate analysis: categorical risk factors for developing PPS during follow-up

In univariate analysis, there were trends suggesting both a higher risk of PPS in adults compared to children (OR 2.37 (95% CI 0.988–5.686, *p* = 0.053)) and a higher odds of PPS with increasing age (per 10 year increment increase:1.233 (95% CI 0.992–1.533, *p* = 0.059)); however, neither of these reached statistical significance (Fig. 2).

**Fig. 3:**
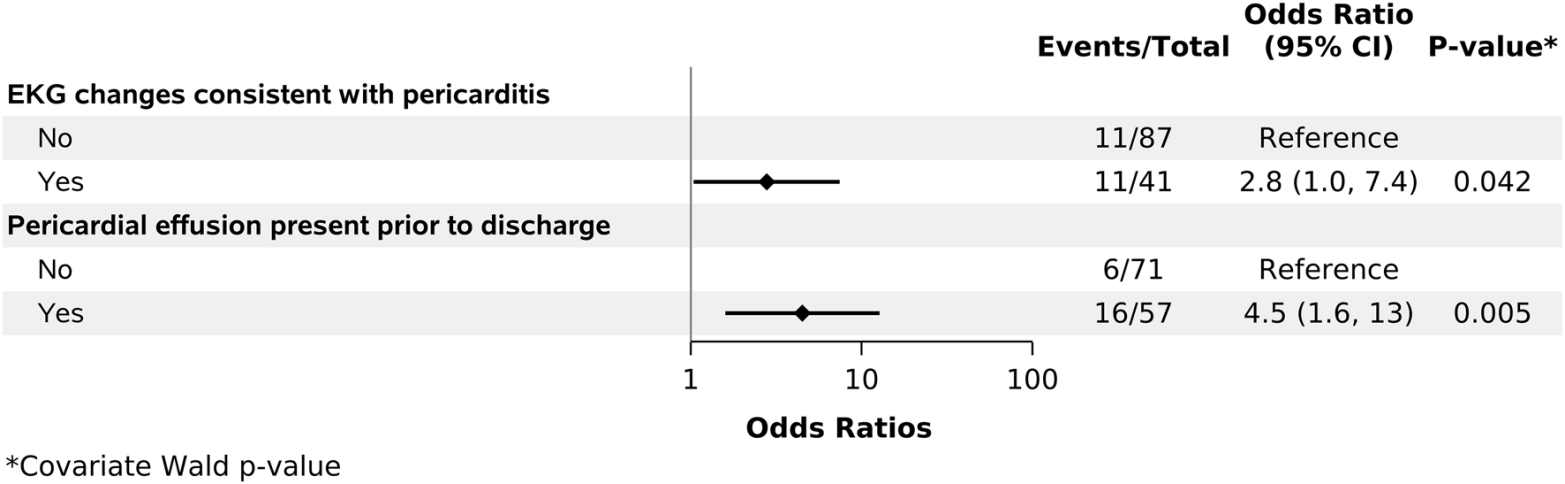
Multivariate analysis risk factorsfor developing PPS during follow-up

The presence of pericardial effusion prior to discharge (72.7% in PPS group vs 38.7% without PPS, *p* < 0.01) and ECG changes suggesting pericarditis (52%in PPS group vs 27.1%without PPS, *p* = 0.02) were both associated with higher odds of PPS development. These associations were maintained in subsequent multivariable analyses (*p* < 0.01 for pericardial effusion and *p* = 0.04 for ECG changes) Other studied variables including steroid use in the OR, number of chest tubes, and blood product administration were not associated with higher odds of PPS (Fig. 3).

## Discussion

In this retrospective review, we studied proposed risk factors that may predict subsequent development of PPS, to find that both early development of post-operative inflammatory ECG changes and early post-operative pericardial effusions may predict subsequent PPS development. A trend was also found as well linking older patients with increased risk of PPS development.

Our study aimed to enhance the understanding of PPS and identify early predictors by utilizing the standardized definition provided by the ESC [1]. By employing this consistent definition, we sought thus more effectively to characterize early risk factors associated with PPS development in perioperative patients who underwent surgically repaired ASD, an approach not consistently used in prior PPS literature. A key hypothesis in our study posited that signs of pericardial inflammation on serial ECGs obtained within the first 72 h of admission would be associated with the development of PPS. This hypothesis is based on the plausible physiological basis of pericardial inflammation and its potential impact on PPS development. Previous studies primarily focused on non-specific ECG findings to identify differences between PPS and non-PPS patients[4, 6, 11]. In contrast, our study involved a blinded comprehensive ECG evaluation by an experienced electrophysiologist to identify changes indicative of inflammation. This approach allowed for a more nuanced understanding of the potential role of ECG findings in predicting PPS. We believe that, because of this approach, we were able to identify a statistical difference in those with PPS, one that was maintained in multivariate analyses.

Earlier investigations exploring the incidence and risk factors of PPS in patients undergoing surgical ASD repairs suggested a disparity in PPS occurrence between adult and pediatric populations. These studies reported that pediatric or younger patients were at a higher risk of developing PPS compared to adults[4, 10]. Importantly, these conclusions were collated from different centers. Our study, conducted at a single center, observed a contrasting trend regarding age groups and the development of PPS. Specifically, our findings indicated that patients who developed PPS had, on average, a higher age compared to those who did not. Although this association did not reach statistical significance in subsequent regression analysis (p = 0.053), it suggests a potential deviation from the previously reported age-related risk pattern, one worthy of further study.

Other than limitations inherent to a single centered retrospective approach, one major limitation included patients lost to follow-up, which may have significantly affected our results. Another concern included the lack of standardized post-operative time frames for obtaining the post-operative ECGs. Better defining timing and consistency of post-operative ECG changes may allow to better understand and strengthen the correlation found. We also were unable to conduct subgroup analysis based on age group, as originally intended, due to limited sample size.

## Conclusion

Our study contributes to the characterization of early risk factors associated with the development of PPS in patients undergoing surgical ASD repairs. By utilizing the standardized ESC definition of PPS and incorporating comprehensive ECG evaluation, early post-operative ECG changes and pericardial effusion development were both identified as potentially powerful early predictors. Although further research is warranted, our findings suggest the importance of considering age as a potential risk factor for PPS. Future studies should build upon these findings and develop strategies for early identification and management of patients at high risk for PPS.
